# Knowledge and Practice of Endocrown Restorations Among Dental Students and Dentists in Sirte, Libya

**DOI:** 10.7759/cureus.66211

**Published:** 2024-08-05

**Authors:** Khadija H Abu Sittah, Mohamed Fattouh, Wahdan A El-Kwatehy, Noora S Berhaim, Laila M Kenawi

**Affiliations:** 1 Department of Prosthodontics, Faculty of Dentistry, Sirte University, Sirte, LBY; 2 Department of Fixed Prosthodontics, Faculty of Dentistry, Cairo University, Cairo, EGY; 3 Department of Pediatric, Dental Public Health and Preventive Dentistry, Faculty of Dentistry, Mansoura University, Mansoura, EGY; 4 Department of Prosthodontics, Faculty of Dentistry and Oral Surgery, University of Tripoli, Tripoli, LBY; 5 Department of Restorative Dentistry, Faculty of Dental Medicine, Umm Al-Qura University, Makkah, SAU; 6 Department of Endodontics, Faculty of Dentistry, Cairo University, Cairo, EGY

**Keywords:** adhesive restoration, surveys and questionnaires, post and core, endodontic restorations, endocrown

## Abstract

Background

Restoring endodontically treated teeth has long posed a challenge for clinicians. The endocrown (EC) is an innovative and conservative restoration designed for teeth with severely damaged coronal structures. ECs offer performance that is equivalent to or even exceeds that of traditional post-core-crown treatments.

Purpose

This web-based cross-sectional survey aimed to evaluate the level of knowledge and practical experience regarding ECs as post-endodontic prostheses among dental students and practitioners in Libya.

Methods

A 22-item structured questionnaire was created using Google Forms and distributed to final-year students, interns, faculty at the College of Dentistry at Sirte University, and practicing dentists in Libya. The sample comprised 290 participants. The questionnaire was divided into three sections: the first assessed demographic variables such as gender, education level, country of graduation, and workplace; the second evaluated knowledge of ECs through 11 questions; and the third focused on EC practice, also comprising 11 questions. Statistical analysis was conducted using IBM SPSS Statistics for Windows, Version 21.0 (Released 2012; IBM Corp., Armonk, NY, USA).

Results

A total of 50.7% of participants indicated that EC restorations are suitable for molar teeth, 41.4% noted that a butt joint finish line is used for EC preparation, and 66.9% preferred all-ceramic materials for ECs. Nearly 72.8% reported that computer-aided design/computer-aided manufacturing technology is employed for EC fabrication. Additionally, 61.7% agreed that EC designs offer higher fracture resistance compared to conventional crowns. Despite this, 64.5% of participants had not cemented an EC in their clinic in recent years. Significant differences in knowledge and practice regarding ECs were observed across various factors, including gender, education level, country of graduation, and workplace.

Conclusion

Most participants demonstrated an acceptable level of knowledge and practical experience with EC restorations. Therefore, incorporating ECs as a major topic in the postgraduate prosthodontics curriculum is recommended.

## Introduction

Restoring endodontically treated teeth has long posed challenges for clinicians. These teeth undergo physiological changes in dentin microstructure and composition, making them susceptible to increased brittleness, reduced retention and stability, compromised substrate adhesion, and, ultimately, prosthesis failure [1]. The endocrown (EC) is a modern, conservative solution designed to address extensively damaged coronal structures in endodontically treated teeth [2]. Introduced by Bindl and Mörmann [3], ECs are indirect ceramic monoblock restorations that gain retention from the pulp chamber. These restorations adhere to the pulp chamber using the micromechanical retention features of its borders [4]. ECs are particularly effective for cases involving significant tooth structure loss in posterior areas [2]. They demonstrate superior performance in molars or teeth with larger pulp chambers [5-7] compared to maxillary [8-10] or mandibular premolars [11-13]. ECs often provide equivalent or even superior outcomes compared to traditional post-core-crown treatments, which involve intra-radicular posts (metal or fiber), cores (composite, amalgam, or glass ionomer), and crowns (ceramic or porcelain fused to metal) [14]. Metal posts, with a higher modulus of elasticity than dentin, can increase the risk of root fractures and failures [15]. In contrast, fiber posts, with mechanical properties similar to dentin, offer a more uniform stress distribution [16]. Compared to traditional posts, cores, and crowns, ECs require less clinical time, simpler preparation, fewer appointments, and offer excellent esthetic results [17]. They are also an ideal option when posts are contraindicated due to short or narrow canals [18].

Several guidelines have been established for the proper preparation of teeth to receive an EC. An occlusal reduction of 2-3 mm is required. It is recommended to use a 90-degree butt-joint margin with a width of 1-1.2 mm [2]. Whenever possible, cervical margins should be placed supragingivally and smoothly transitioned internally to the flat pulpal floor. Additionally, an occlusal divergence of 5-7 degrees is necessary for the endodontic access hole and coronal pulp cavity, ensuring continuous preparation. The depth of the pulp chamber should be adequate to provide retention and resistance [9,19,20]. EC restorations can be fabricated using either computer-aided design/computer-aided manufacturing (CAD/CAM) systems or conventional heat-pressing ceramics. Currently, CAD/CAM technology is preferred for its ability to deliver high-quality restorations with reduced chairside time [18].

Dental students and dentists must be well informed about various treatment options to meet patient expectations and ensure the long-term success of prosthetic restorations. To date, no research has specifically evaluated the knowledge and practice of EC restorations in Libya. Therefore, this web-based cross-sectional survey was conducted to assess the current state of knowledge and practice regarding ECs as a post-endodontic prosthesis among Libyan dental students and dentists.

## Materials and methods

The study was approved by the Biomedical Research Ethical Committee of the National Center for Disease Control (NCDC), Ministry of Health, Libya, and conducted in accordance with the principles of the Helsinki Declaration [21].

A structured questionnaire was developed using Google Forms and was accessible via PCs, mobiles, or tablets. It was distributed from December 2023 to January 2024 to assess participants’ knowledge and practice regarding EC restorations. The questionnaire was sent to all final-year students, interns, and faculty at the College of Dentistry, Sirte University, as well as to dentists across Libya. All participants provided informed consent.

The 22-item closed questionnaire was based on the 2023 study by Al Moaleem et al., with minor modifications [22]. Respondents selected one answer per question using checkboxes, and each participant was allowed to respond only once. All questions were mandatory, and Google Forms recorded the responses.

The questionnaire consisted of three sections (Appendix 1). The first section collected demographic information, including gender, level of education, country of graduation, and workplace. The second section assessed knowledge of ECs with 11 questions, while the third section focused on EC practice with another 11 questions.

Study design

This cross-sectional study aimed to establish a baseline level of awareness and management of endodontically treated teeth restored with ECs among oral health practitioners in Sirte, Libya.

Ethical consideration

The study adhered to the guidelines set forth by the World Medical Association Declaration of Helsinki. Ethical approval was granted by the Biomedical Research Ethics Committee of the NCDC, Ministry of Health, Libya (approval number NBC: 002. H-23.22). Informed consent was obtained from all participants prior to the study.

Participants

A questionnaire survey was conducted among dental students, interns, faculty members, and practitioners in public hospitals and private clinics in Sirte, Libya. The sample size was calculated according to the formula used for the whole population: n = Z2 × p × q / e2, where n = the required sample size, q = 1 - p, with a 95% level of confidence and sample error ±5%. Assuming q = 0.5 to achieve the maximum sample size, a total of 290 participants were surveyed.

The questionnaire, designed to assess dentists’ knowledge and practice of ECs, was distributed from December 2023 to April 2024. It included questions relevant to understanding EC restorations, ensuring the study's goals were met. The survey targeted undergraduate students, general dentists, specialists, and consultants working in both academic and clinical settings.

To assess the reliability of the questionnaire, the test-retest method was employed. Ten participants from the Faculty of Dentistry, Sirte University who were not involved in the main study completed the questionnaire twice within a two-week interval. Pearson’s correlation coefficient (Pearson’s r) indicated a significant stability coefficient, suggesting good test-retest reliability. Internal consistency was measured using Cronbach’s alpha, with a value of α = 0.767, indicating adequate internal consistency between items [23].

Statistical analysis

Statistical analysis was conducted using IBM SPSS Statistics for Windows, Version 21.0 (IBM Corp., Armonk, NY, USA), and results were visually presented using GraphPad Prism version 8 (GraphPad Software, Inc., San Diego, CA, USA). Nominal and categorical variables were described using simple descriptive statistics, including counts and percentages. The significance level was set at 0.05. Comparisons of results between groups defined by participants' characteristics were performed using the chi-square test.

## Results

The survey included 290 participants, with 235 females (81%) and 55 males (19%). Of these participants, 51 (17.6%) were undergraduates, 59 (20.3%) were interns, 97 (33.4%) had graduated within the past five years, and 83 (28.6%) had five or more years of experience. Regarding their graduation country, 258 (89%) of the participants graduated from Libya. In terms of the workplace, 111 (38.3%) were employed at universities, 77 (26.6%) in the private sector, and 65 (22.4%) in the government sector (Table 1).

**Table 1:** Distribution of subjects according to gender, level of education, country of graduation, and working place (demographic parameters)

Variables		Frequency	Percent
Gender	Female	235	81
	Male	55	19
Level of education	Undergraduate	51	17.6
	Intern	59	20.3
	Graduated within the past five years	97	33.4
	Graduated with five years or more of experience	83	28.6
Graduation country	Libya	258	89
	Other countries	32	11
Workplace	University	111	38.3
	Private	77	26.6
	Governmental	65	22.4
	Unemployed	37	12.8
Total		290	100

Participants’ knowledge of ECs showed that 150 (51.7%) learned about ECs during college, and 147 (50.7%) recognized their indication for molar teeth. A total of 227 (78.3%) understood ECs as a minimally invasive solution that conserves tooth structure. A total of 120 (41.4%) noted the use of a butt joint finish line for preparation, while 194 (66.9%) preferred all-ceramic materials. Moreover, 211 (72.8%) identified CAD/CAM technology for fabrication, and 179 (61.7%) acknowledged ECs’ superior fracture resistance over conventional crowns. Furthermore, 128 (44.1%) endorsed a 2 mm pulp chamber extension, and 198 (68.3%) believed irregular pulp chamber floors increased failure risk. A total of 122 (42.1%) feared increased tooth fractures with ECs, yet 198 (68.3%) saw ECs as a suitable alternative to traditional post, core, and crown systems (Table 2).

**Table 2:** Distribution of subjects based on their EC knowledge CAD/CAM, computer-aided design/computer-aided manufacturing; EC, endocrown

Question	Frequency	Percent
Q1: From where did you get the information about EC?		
College	150	51.7
Media	68	23.4
Combination	35	12.1
Email	11	3.8
workshop	26	9
Q2: EC restorations are indicated in:		
Molar	147	50.7
Molar and premolar	78	26.9
All teeth	49	16.9
I don’t know	16	5.5
Q3: The goal of using EC is to achieve minimal invasive preparations and preserve the existing tooth structure.		
True	227	78.3
False	40	13.8
I don’t know	23	7.9
Q4: Which type of finish line is used during EC preparation?		
Butt joint	120	41.4
Chamfer	48	16.6
Shoulder	65	22.4
I don’t know	57	19.7
Q5: The material of choice for ECs is ceramic.		
Yes	194	66.9
No	68	23.4
I don’t know	28	9.7
Q6: ECs are fabricated using:		
CAD/CAM technique	211	72.8
Pressable technique	37	12.8
I don’t know	42	14.5
Q7: The fracture resistance of EC is higher than that of conventional crowns.		
True	179	61.7
False	75	25.9
I don’t know	36	12.4
Q8: The ideal extension of EC in the pulp chamber is:		
2 m	128	44.1
3 m	72	24.8
4 m	36	12.4
I don’t know	54	18.6
Q9: If the floor of the pulp chamber is irregular, the failure of EC is increased.		
True	198	68.3
False	42	14.5
I don’t know	50	17.2
Q10: Does EC increase the rate of tooth fracture?		
Always	26	9
Sometimes	122	42.1
Never	32	31
I don’t know	52	17.9
Q11: Can EC be a substitute for a conventional post, core, and crown?		
Yes	198	68.3
No	48	16.6
I don’t know	44	15.2

Regarding the practice of ECs in their clinics, 188 (64.5%) of participants reported not having cemented ECs in recent years. Only 125 (43.1%) consistently used rubber dams during EC cementation, while 150 (51.7%) always utilized X-rays to verify the extension of ECs in the pulp chamber. The predominant cause of failure, identified by 190 (65.5%), was the debonding of ECs. For tooth preparation, 177 (61%) found it no more difficult than conventional post, core, and crown preparations, and 165 (56.9%) felt that EC preparations and impression-making were not more time-consuming. Additionally, 187 (64.5%) believed that the presence of an enamel margin enhanced fracture resistance and bonding strength (Table 3).

**Table 3:** Distribution of subjects based on their EC practice EC, endocrown

Question	Frequency	Percent
Q12: How many ECs did you cement in the clinic in previous years?		
None	188	64.5
Less than 5	56	19.7
Between 5 and 10	30	10.3
More than 10	16	5.5
Q13: How often do you use a rubber dam during EC cementation?		
Always	125	43.1
Sometimes	51	17.6
Never	114	39.3
Q14: How often do you use a radiograph to check the EC extension in the pulp chamber?		
Always	150	51.7
Sometimes	42	14.5
Never	98	33.8
Q15: The most common “failure mode” of EC is debonding.		
Yes	190	65.5
No	27	9.3
I don’t know	73	25.2
Q16: Tooth preparation steps to receive EC are more difficult than post, core, and crown.		
Yes	74	25.5
No	177	61
I don’t know	39	13.4
Q17: Tooth preparation and impression to receive EC are more time-consuming than post, core, and crown.		
Yes	73	25.2
No	165	56.9
I don’t know	52	17.9
Q18: Fracture resistance and bonding strength can be increased by the presence of an enamel margin.		
Yes	187	64.5
No	39	13.4
I don’t know	64	22

Using the chi-square test, no significant association was found between knowledge and gender (p > 0.05) (Table 4). However, significant differences in responses were observed based on education level for questions Q1, Q6, Q8, Q10, and Q11 (p < 0.05) (Table 5). Differences were also significant for the country of graduation concerning questions Q1, Q4, Q5, and Q8 (Table 6). Additionally, significant variations were noted in responses related to the workplace of participants for questions Q1, Q2, and Q3 (Table 7).

**Table 4:** Knowledge association with gender CAD/CAM, computer-aided design/computer-aided manufacturing; EC, endocrown

Variables		Gender		
		Male, N (%)	Female, N (%)	p-value
Q1: From where did you get information about EC?	College	21 (38.2)	129 (54.9)	0.092
	Media	18 (32.7)	50 (21.3)	
	Combination	7 (12.7)	28 (11.9)	
	Email	1 (1.8)	10 (4.30)	
	Workshop	8 (14.5)	18 (7.7)	
Q2: EC restorations are indicated in:	Molar	29 (52.7)	118 (50.2)	0.22
	Molar and premolar	19 (34.5)	59 (25.1)	
	All teeth	5 (9.1)	44 (18.7)	
	I don’t know	2 (3.6)	14 (6.0)	
Q3: The goal of using EC is to achieve minimal invasive preparations and preserve existing tooth structure.	True	43 (78.2)	184 (78.3)	0.968
	False	8 (14.5)	32 (13.6)	
	I don’t know	4 (7.3)	19 (8.1)	
Q4: Which type of finish line is used during EC preparation?	Butt joint	24 (43.6)	96 (40.9)	0.314
	Chamfer	10 (18.2)	38 (16.2)	
	Shoulder	15 (27.3)	50 (21.3)	
	I don’t know	6 (10.9)	51 (21.7)	
Q5: The material of choice for EC is all ceramic.	Yes	38 (69.1)	156 (66.4)	0.929
	No	12 (21.8)	56 (23.8)	
	I don’t know	5 (9.1)	23 (9.8)	
Q6: ECs are fabricated using:	CAD/CAM	43 (78.2)	168 (71.5)	0.237
	Pressable	8 (14.5)	38 (16.2)	
	I don’t know	4 (7.3)	29 (12.3)	
Q7: The fracture resistance of EC is higher than that of conventional crowns.	True	40 (72.7)	139 (59.1)	0.148
	False	9 (16.4)	66 (28.1)	
	I don’t know	6 (10.9)	30 (12.8)	
Q8: The ideal extension of EC in the pulp chamber is:	2 m	23 (41.8)	105 (44.7)	0.983
	3 m	14 (25.5)	58 (24.7)	
	4 m	7 (12.7)	43 (18.3)	
	I don’t know	11 (20.0)	29 (12.3)	
Q9: If the floor of the pulp chamber is irregular, the failure of EC is increased.	True	40 (72.7)	158 (67.2)	0.379
	False	9 (16.4)	33 (14.0)	
	I don’t know	6 (10.9)	44 (18.7)	
Q10: Does EC increase the rate of tooth fracture?	Always	3 (5.5)	23 (9.8)	359
	Sometimes	20 (36.4)	102 (43.4)	
	Never	22 (40.0)	68 (28.9)	
	I don’t know	10 (18.2)	42 (17.9)	
Q11: Can EC be a substitute for a conventional post, core, and crown?	Yes	38 (69.1)	160 (68.1)	0.826
	No	10 (18.2)	38 (16.2)	
	I don’t know	7 (12.7)	37 (15.7)	

**Table 5:** Knowledge association with education level CAD/CAM, computer-aided design/computer-aided manufacturing; EC, endocrown

Variables		Level of education				
		Undergraduate, N (%)	Intern, N (%)	Graduated within the past five years, N (%)	Graduated with five years or more of experience, N (%)	p-value
Q1: From where did you get information about EC?	College	45 (88.2)	41 (69.5)	47 (48.5)	17 (20.5)	0
	Media	4 (7.8)	6 (10.2)	26 (26.8)	32 (38.6)	
	Combination	2 (39)	6 (10.2)	16 (16.5)	13 (15.7)	
	Email	0 (0.0)	4 (6.8)	2 (2.1)	5 (6.0)	
	Workshop	0 (0.0)	2 (3.4)	6 (6.2)	16 (19.3)	
Q2: EC restorations are indicated in:	Molar	27 (52.9)	34 (57.6)	49 (50.5)	37 (44.6)	0.208
	Molar and premolar	10 (19.6)	14 (23.7)	29 (29.9)	25 (30.1)	
	All teeth	13 (25.5)	9 (15.3)	15 (15.5)	12 (14.5)	
	I don’t know	1 (2.0).	2 (3.4)	4 (4.1)	9 (10.8)	
Q3: The goal of using EC is to achieve minimal invasive preparations and preserve existing tooth structure.	True	39 (76.5)	45 (76.3)	78 (80.4)	65 (78.3)	0.829
	False	9 (17.6)	9 (15.3)	13 (13.4)	9 (10.8)	
	I don’t know	3 (5.9)	5 (8.5)	6 (6.2)	9 (10.8)	
Q4: Which type of finish line is used during EC preparation?	Butt joint	21 (41.2)	31(52.5)	39 (40.2)	29 (34.9)	0.061
	Chamfer	10 (19.6)	8 (13.6)	18 (18.6)	12 (14.5)	
	Shoulder	16 (31.4)	13 (22.0)	19 (19.6)	17 (20.5)	
	I don’t know	4 (7.8)	7 (11.9)	21 (21.6)	25 (30.1)	
Q5: The material of choice for EC is all ceramic.	Yes	42 (82.4)	37 (62.7)	59 (60.8)	56 (67.5)	0.113
	No	8 (15.7)	16 (27.1)	28 (28.9)	16 (19.3)	
	I don’t know	1 (2.0)	6 (10.2)	10 (10.3)	11 (13.3)	
Q6: ECs are fabricated using:	CAD/CAM	42 (82.4)	47 (79.7)	70 (72.2)	52 (62.9)	0.011
	Pressable	0 (0.0)	8 (13.6)	11 (11.3)	14 (16.9)	
	I don’t know	9 (17.6)	4 (6.8)	16 (16.5)	17 (20.5)	
Q7: The fracture resistance of EC is higher than that of conventional crowns.	True	33 (64.7)	39 (66.1)	54 (55.7)	53 (63.9)	0.25
	False	15 (29.4)	14 (23.7)	24 (24.7)	22 (26.5)	
	I don’t know	3 (5.9)	6 (10.2)	19 (19.6)	8 (9.6)	
Q8: The ideal extension of EC in the pulp chamber is:	2 m	25(49)	30 (50.8)	44 (45.4)	29 (34.9)	0.011
	3 m	16 (31.4)	13 (22.0)	18 (18.6)	25 (30.1)	
	4 m	4 (7.80)	10 (17.0)	19 (19.6)	24 (32.5)	
	I don’t know	6 (11.8)	6 (10.2)	16 (16.5)	5 (6.0)	
Q9: If the floor of the pulp chamber is irregular, the failure of EC is increased.	True	32 (62.7)	42 (71.2)	69 (71.1)	55 (66.3)	0.757
	False	11 (21.6)	8 (13.6)	12 (12.4)	11 (13.3)	
	I don’t know	8 (15.7)	9 (15.3)	16 (16.5)	17(20.5)	
Q10: Does EC increase the rate of tooth fracture?	Always	9 (17.6)	3 (5.1)	5 (5.2)	9 (10.8)	0.009
	Sometimes	28 (54.9)	27 (45.8)	42 (43.3)	25 (30.1)	
	Never	8 (15.7)	15 (25.4)	35 (35.1)	32 (38.6)	
	I don’t know	6 (11.80	14 (23.7)	15 (15.5)	17 (20.5)	
Q11: Can EC be a substitute for a conventional post, core, and crown?	Yes	43 (84.3)	39 (66.1)	57 (58.8)	59 (71.1)	0.022
	No	6 (11.8)	13 (22.0)	17 (17.5)	12 (14.5)	
	I don’t know	2 (3.9)	7 (11.9)	23 (37.7)	12 (14.5)	

**Table 6:** Knowledge association with country of graduation CAD/CAM, computer-aided design/computer-aided manufacturing; EC, endocrown

Variables		Graduation country		
		Libya, N (%)	Others, N (%)	p-value
Q1: From where did you get information about EC?	College	138 (53.5)	12 (37.5)	0.017
	Media	52 (20.2)	16 (50.0)	
	Combination	33 (12.8)	2 (6.3)	
	Email	11 (4.3)	0 (0.0)	
	Workshop	24 (9.3)	2 (6.3)	
Q2: EC restorations are indicated in:	Molar	137 (53.1)	10 (31.1)	0.513
	Molar and premolar	66 (25.6)	12 (37.5)	
	All teeth	41 (15.9)	8 (25.0)	
	I don’t know	14 (5.4)	2 (6.3)	
Q3: The goal of using EC is to achieve minimal invasive preparations and preserve existing tooth structure.	True	207 (80.2)	20 (62.5)	0.155
	False	33 (12.8)	7 (21.9)	
	I don’t know	18(7.0)	5 (15.6)	
Q4: Which type of finish line is used during EC preparation?	Butt joint	116 (45.0)	4 (12.5)	0.002
	Chamfer	39 (15.1)	9 (28.1)	
	Shoulder	58 (22.5)	7 (21.9)	
	I don’t know	45 (17.4)	12 (37.5)	
Q5: The material of choice for EC is all ceramic.	Yes	183 (709.9)	11 (34.4)	0.004
	No	53(20.5)	15 (46.9)	
	I don’t know	22 (8.5)	6 (18.8)	
Q6: ECs are fabricated using:	CAD/CAM	189 (73.3)	22 (68.8)	0.395
	Pressable	34 (13.2)	8 (25.0)	
	I don’t know	35 (13.6)	2 (6.3)	
Q7: The fracture resistance of EC is higher than that of conventional crowns.	True	165 (64.0)	14 (43.8)	0.132
	False	65 (25.2)	10 (31.3)	
	I don’t know	28 (10.9)	8 (25.0)	
Q8: The ideal extension of EC in the pulp chamber is	2 m	114 (44.2)	14 (43.8)	0.031
	3 m	69 (26.7)	3 (9.4)	
	4 m	26 (10.1)	10 (31.3)	
	I don’t know	49 (19.0)	5 (15.6)	
Q9: If the floor of the pulp chamber is irregular, the failure of EC is increased.	True	173 (67.1)	25 (78.1)	0.852
	False	39 (15.1)	3 (9.4)	
	I don’t know	46 (17.8)	4 (12.5)	
Q10: Does EC increase the rate of tooth fracture?	Always	22 (8.5)	4 (12.5)	0.308
	Sometimes	106 (41.1)	16 (50.0)	
	Never	82 (31.8)	8 (25.0)	
	I don’t know	48 (18.6)	4 (12.5)	
Q11: Can EC be a substitute for a conventional post, core, and crown?	Yes	180 (69.8)	18 (56.3)	0.139
	No	43 (16.7)	5 (15.6)	
	I don’t know	35 (13.6)	9 (28.1)	

**Table 7:** Knowledge association with place of work CAD/CAM, computer-aided design/computer-aided manufacturing; EC, endocrown

Variables		Workplace				
		University, N (%)	Private, N (%)	Unemployed, N (%)	Governmental, N (%)	p-value
Q1: From where did you get information about EC?	College	75 (67.6)	26 (33.6)	31 (83.8)	18 (27.7)	0
	Media	10 (9.0)	24 (31.2)	3 (8.1)	31 (47.7)	
	Combination	15 (13.5)	11 (14.3)	1 (2.7)	8 (12.3)	
	Email	3 (2.7)	3 (3.9)	2 (5.4)	3 (4.6)	
	Workshop	8 (7.2)	13 (16.9)	0 (0.00)	5 (7.7)	
Q2: EC restorations are indicated in:	Molar	59 (53.2)	34 (44.2)	25 (67.6)	29 (44.6)	0.039
	Molar and premolar	28 (25.2)	30 (39.0)	4 (10.8)	16 (24.6)	
	All teeth	21 (18.9)	8 (10.4)	6 (17.2)	14 (21.5)	
	I don’t know	3 (2.7)	5 (6.5)	2 (5.2)	6 (9.2)	
Q3: The goal of using EC is to achieve minimal invasive preparations and preserve existing tooth structures.	True	89 (80.2)	64 (83.1)	28 (75.7)	46 (70.8)	0.004
	False	16 (14.4)	12 (15.6)	6 (16.2)	6 (9.2)	
	I don’t know	6(5.4)	1 (1.3)	3 (8.1)	13 (20.0)	
Q4: Which type of finish line is used during EC preparation?	Butt joint	51 (45.9)	33 (42.9)	18 (48.6)	18 (27.7)	0.059
	Chamfer	16 (14.4)	9 (11.7)	9 (24.3)	14 (21.5)	
	Shoulder	28 (25.2)	15 (19.5)	7 (18.9)	15 (23.1)	
	I don’t know	16 (14.4)	20 (26.0)	3 (8.1)	18 (27.7)	
Q5: The material of choice for EC is all ceramic.	Yes	72 (64.9)	54 (70.1)	30 (81.1)	38 (58.5)	0.182
	No	31 (27.9)	15 (19.5)	5 (13.5)	17 (26.2)	
	I don’t know	8 (7.2)	8 (10.4)	2 (5.4)	10 (15.4)	
Q6: ECs are fabricated using:	CAD/CAM	85 (76.6)	55 (71.4)	26 (70.3)	45 (69.2)	0.285
	Pressable	17 (15.3)	7 (9.1)	6 (16.2)	12 (18.5)	
	I don’t know	9 (8.1)	15 (19.5)	5 (13.5)	8 (12.3)	
Q7: The fracture resistance of EC is higher than that of conventional crowns.	True	73 (65.8)	48 (62.3)	24 (64.9)	34 (52.3)	0.681
	False	27 (24.3)	18 (23.4)	9 (24.3)	21 (32.3)	
	I don’t know	11 (9.9)	11 (14.3)	4 (10.8)	10 (15.4)	
Q8: The ideal extension of EC in the pulp chamber is:	2 m	52 (46.8)	36 (46.8)	17 (45.9)	23 (35.4)	0.579
	3 m	30 (27.0)	15 (19.5)	11 (29.7)	16 (24.6)	
	4 m	16 (14.4)	8 (10.4)	5 (13.5)	11 (16.9)	
	I don’t know	13 (11.7)	18 (23.4)	4 (10.8)	15 (23.1)	
Q9: If the floor of the pulp chamber is irregular, the failure of EC is increased.	True	80 (72.1)	51(66.2)	25 (67.6)	42 (64.6)	0.755
	False	16 (14.4)	10 (13.0)	4 (10.8)	12(18.5)	
	I don’t know	15 (13.5)	16 (20.8)	8 (21.6)	11 (16.9)	
Q10: Does EC increase the rate of tooth fracture?	Always	10 (9.0)	4 (5.2)	5 (13.5)	7 (10.8)	0.292
	Sometimes	48 (43.2)	25 (32.5)	19 (51.4)	30 (46.2)	
	Never	31 (27.9)	32 (41.6)	9 (24.3)	18 (27.7)	
	I don’t know	22 (19.8)	16 (20.8)	4 (10.8)	10 (15.4)	
Q11: Can EC be a substitute for a conventional post, core, and crown?	Yes	75 (67.6)	53 (68.8)	26 (70.3)	44 (67.7)	0.983
	No	20 (18.0)	11 (14.3)	5 (13.5)	12 (18.5)	
	I don’t know	16 (14.4)	13 (16.9)	6 (16.2)	9 (13.8)	

There was no significant association between practice and gender or country of graduation (p > 0.05) (Table 8, Table 9). Significant differences were observed in practice related to the level of education for questions Q13 (p = 0.001), Q14 (p = 0.006), and Q15 (p = 0.021) (Table 10). Regarding the workplace, a significant difference was found for question Q16 (p = 0.019), while no significant differences were observed for other questions (p > 0.05) (Table 11).

**Table 8:** Practice association with gender EC, endocrown

Variables		Gender		
		Male, N (%)	Female, N (%)	p-value
Q12: How many ECs did you cement in the clinic in previous years?	None	29 (52.7)	158(67.2)	0.168
	Less than five	15 (27.3)	42 (17.9)	
	Between 5 and 10	6 (10.9)	24 (10.2)	
	More than 10	5 (9.1)	11 (4.7)	
Q13: How often do you use a rubber dam during EC cementation?	Always	29 (52.7)	96 (40.9)	0.065
	Sometimes	12 (21.8)	39 (16.6)	
	Never	14 (25.5)	100(42.6)	
Q14: How often do you use a radiograph to check the EC extension in the pulp chamber?	Always	36 (65.5)	114 (48.5)	0.076
	Sometimes	6 (10.9)	36 (15.3)	
	Never	13 (23.6)	85 (36.2)	
Q15: The most common “failure mode” of EC is debonding.	Yes	39 (70.9)	151 (64.3)	0.599
	No	5 (9.1)	22 (9.4)	
	I don’t know	11 (20.0)	62 (26.4)	
Q16: Tooth preparation steps to receive EC are more difficult than post, core, and crown.	Yes	13 (23.6)	61 (26.0)	0.922
	No	34 (61.8)	143 (60.9)	
	I don’t know	8 (14.5)	31 (13.2)	
Q17: Tooth preparation and impression to receive EC are more time-consuming than post, core, and crown.	Yes	11 (20.0)	62 (26.4)	0.603
	No	34 (61.8)	131 (55.7)	
	I don’t know	10 (18.2)	42 (17.9)	
Q18: Fracture resistance and bonding strength can be increased by the presence of an enamel margin.	Yes	40 (72.7)	147 (62.6)	0.101
	No	8 (14.5)	31 (13.2)	
	I don’t know	7 (12.7)	57 (24.3)	

**Table 9:** Practice association with country of graduation EC, endocrown

Variables		Graduation country		
		Libya	Others	p-value
Q12: How many ECs did you cement in the clinic in previous years?	None	169 (65.5)	18 (56.3)	0.497
	Less than five	49 (19.0)	8 (25.0)	
	Between 5 and 10	24 (9.3)	6 (18.8)	
	More than 10	16 (6.2)	0 (0.00)	
Q13: How often do you use a rubber dam during EC cementation?	Always	110 (42.6)	15 (46.9)	0.3
	Sometimes	42 (16.3)	9 (28.1)	
	Never	106 (41.1)	8 (25.0)	
Q14: How often do you use a radiograph to check the EC extension in the pulp chamber?	Always	131 (50.8)	19 (59.4)	0.748
	Sometimes	37 (14.3)	5 (15.6)	
	Never	90 (34.9)	8 (25.0)	
Q15: The most common “failure mode” of EC is debonding.	Yes	171 (66.3)	19 (59.4)	0.61
	No	22 (8.5)	5 (15.6)	
	I didn’t know	65 (25.2)	8 (25.0)	
Q16: Tooth preparation steps to receive EC are more difficult than post, core, and crown.	Yes	63 (24.4)	11 (34.4)	0.088
	No	163 (63.2)	14 (43.8)	
	I didn’t know	32 (12.4)	7 (21.9)	
Q17: Tooth preparation and impression to receive EC are more time-consuming than post, core, and crown.	Yes	62 (24.0)	11 (34.4)	0.085
	No	154 (59.7)	11 (34.4)	
	I didn’t know	42 (16.3)	10 (31.3)	
Q18: Fracture resistance and bonding strength can be increased by the presence of an enamel margin.	Yes	164 (63.6)	23 (71.9)	0.981
	No	37 (14.3)	2 (6.3)	
	I didn’t know	57 (22.2)	7 (21.9)	

**Table 10:** Practice association with education level EC, endocrown

Variables		Level of education				
		Undergraduate, N (%)	Intern, N (%)	Graduated within the past five years, N (%)	Graduated with five years or more of experience, N (%)	p-value
Q12: How many ECs did you cement in the clinic in previous years?	None	38 (74.5)	39 (66.1)	56 (57.7)	54 (65.1)	0.268
	Less than 5	6 (11.8)	11 (18.6)	23 (23.7)	17 (20.5)	
	Between 5 and 10	2 (3.90)	8 (13.6)	13 (13.4)	7 (8.4)	
	More than 10	5 (9.8)	1 (1.7)	5 (5.2)	5 (6.0)	
Q13: How often do you use a rubber dam during EC cementation?	Always	35 (68.6)	23 (39.0)	37 (38.1)	30 (36.1)	0.001
	Sometimes	3 (5.9)	12 (20.3)	24 (24.7)	12 (14.5)	
	Never	13 (25.5)	24 (40.7)	36 (37.1)	41 (49.4)	
Q14: How often do you use a radiograph to check the EC extension in the pulp chamber?	Always	34 (66.70	33 (55.9)	54 (55.7)	29 (34.9)	0.006
	Sometimes	8 (16.7)	9 (15.3)	12 (12.4)	13 (15.7)	
	Never	9 (17.6)	17 (28.8)	31 (32.0)	41 (49.1)	
Q15: The most common “failure mode” of EC is debonding.	Yes	40 (78.4)	43 (72.9)	62 (63.9)	45 (54.2)	0.021
	No	3 (5.9)	6 (10.2)	12 (12.4)	6 (7.2)	
	I didn’t know	8 (15.7)	10 (16.9)	23 (23.7)	32 (38.6)	
Q16: Tooth preparation steps to receive EC are more difficult than post, core, and crown.	Yes	17 (33.3)	18 (30.5)	27 (27.8)	12 (14.5)	0.055
	No	30 (58.8)	35 (59.3)	59 (60.8)	53 (63.9)	
	I didn’t know	4 (7.8)	6 (10.2)	11 (11.3)	18 (21.7)	
Q17: Tooth preparation and impression to receive EC are more time-consuming than post, core, and crown.	Yes	20 (39.2)	12 (20.3)	22 (22.7)	19 (22.9)	0.165
	No	24 (47.1)	39 (66.1)	57 (58.8)	45 (54.2)	
	I didn’t know	7 (13.7)	8 (13.6)	18 (18.6)	19 (22.9)	
Q18: Fracture resistance and bonding strength can be increased by the presence of an enamel margin.	Yes	38 (74.5)	44 (74.6)	59 (60.8)	46 (55.4)	0.087
	No	7 (13.7)	8 (13.6)	16 (16.5)	8 (9.6)	
	I didn’t know	6 (11.8)	7 (11.9)	22 (22.7)	29 (34.9)	

**Table 11:** Practice association with place of work EC, endocrown

Variables		Workplace				
		University, N (%)	Private, N (%)	Unemployed, N (%)	Governmental, N (%)	p-value
Q12: How many ECs did you cement in the clinic in previous years?	None	75 (67.6)	44 (57.1)	25 (67.6)	43 (66.2)	0.153
	Less than 5	24 (21.6)	18 (23.4)	5 (13.5)	10 (15.4)	
	Between 5 and 10	8 (7.2)	7 (29.1)	4 (10.8)	11 (16.9)	
	More than 10	4 (3.6)	8 (10.4)	3 (8.1)	1 (1.5)	
Q13: How often do you use a rubber dam during EC cementation?	Always	50 (45.0)	32 (41.6)	22 (59.5)	21 (32.3)	0.122
	Sometimes	17 (15.3)	18 (23.4)	3 (8.1)	13 (20.0)	
	Never	44 (39.6)	27 (35.1)	12 (32.4)	31 (47.7)	
Q14: How often do you use a radiograph to check the EC extension in the pulp chamber?	Always	62 (55.9)	36 (46.8)	20 (54.1)	32 (49.2)	0.513
	Sometimes	16 (14.4)	13 (16.9)	7 (18.9)	6 (9.2)	
	Never	33 (29.7)	28 (36.4)	10 (27.0)	27 (41.5)	
Q15: The most common “failure mode” of EC is debonding.	Yes	74 (66.7)	52 (67.5)	26 (70.3)	38 (58.5)	0.513
	No	11 (9.9)	4 (5.2)	5 (13.5)	7 (10.8)	
	I didn’t know	26 (23.4)	21 (27.3)	6 (16.2)	20 (30.8)	
Q16: Tooth preparation steps to receive EC are more difficult than post, core, and crown.	Yes	29 (26.1)	19 (24.7)	11 (29.7)	15 (23.1)	0.019
	No	75 (67.6)	49 (63.6)	20 (54.1)	33 (50.8)	
	I didn’t know	7 (6.3)	9 (11.7)	6 (16.2)	17 (26.2)	
Q17: Tooth preparation and impression to receive EC are more time-consuming than post, core, and crown.	Yes	30 (27.0)	17 (22.1)	8 (21.6)	18 (27.7)	451
	No	67 (60.4)	46 (59.7)	21 (56.8)	31 (47.7)	
	I didn’t know	14 (12.6)	14 (18.2)	8 (21.6)	16 (24.6)	
Q18: Fracture resistance and bonding strength can be increased by the presence of an enamel margin.	Yes	73 (65.8)	47 (61.0)	27 (73.0)	40 (61.5)	0.771
	No	16 (14.4)	12 (15.6)	5 (13.5)	6 (9.2)	
	I didn’t know	22 (19.6)	18 (23.4)	5 (13.5	19 (29.2)	

## Discussion

ECs are employed to restore extensively damaged, endodontically treated teeth. They depend on the remaining tooth structure for support, with retention provided by the pulp chamber and surrounding dentine [7]. EC restorations present an effective and conservative approach for restoring severely destructed endodontically treated teeth. However, they require careful case selection, precise preparation, and accurate cementation techniques to ensure long-term success. This cross-sectional study aimed to assess the level of knowledge, awareness, and beliefs about ECs among dental students and practitioners in Sirte, Libya. It found that approximately half of the dentists obtained their information about ECs from college (51.7%), while 23.4% sourced their knowledge from the media. The remainder came from a combination of methods, including workshops and emails.

In our study, half of the participants believed that ECs are used exclusively for molars, while 26.9% reported using them for both premolars and molars. Sevimli et al. [17] suggested that ECs can effectively restore endodontically treated incisors, premolars, and molars. However, Bindl et al. [24] concluded that a small pulp chamber in premolars can negatively impact resin-cement adhesion. Additionally, Lander and Dietschi [25] found that the greater length compared to the width of most premolars increases the risk of fracture and displacement of ECs. Therefore, it is recommended to limit the use of ECs to molar teeth, particularly those with shorter crowns and narrow or calcified root canals [23,26].

Preparing teeth to receive ECs is a straightforward procedure that is not time-consuming. In our study, most participants (61%) reported that EC procedures are not difficult. Additionally, 56.9% found that tooth preparation and impression-making for ECs are less time-consuming compared to conventional post, core, and crown procedures. Deepak and Nivedhitha [27] conducted a cross-sectional study on dentists in Chennai, India, and concluded that ECs can serve as a practical alternative for restoring mutilated teeth. The preparation for ECs involves creating a 1.0-1.2 mm circumferential butt margin with a central retention cavity inside the flat pulp chamber. This design does not rely on support from the root canals but is constructed as a single monoblock unit that integrates both the crown and core [3,28]. A butt-joint margin, also known as a cervical sidewalk, is a 90-degree circumferential enamel margin with a width of 1-2 mm [19,29]. In our survey, 41.4% of participants indicated that this is their preferred margin for EC preparation. Compared to the shoulder margin with the ferrule effect, the butt-joint margin is simpler and provides better internal adaptation and marginal integrity [30].

The enamel margin improves bonding and enhances the strength of the restoration by providing a horizontal surface that can withstand compressive stresses [29,31]. In our study, 66.9% of participants reported that the materials used for ECs are ceramics, specifically monolithic zirconia or lithium disilicate. Lithium disilicate is often preferred due to its adhesion properties, imparted by its silica content and its ability to be etched [32,33]. Composite resin materials, on the other hand, are weaker, less durable, and prone to microleakage [34,35]. Therefore, careful consideration of the preparatory design and material selection is essential during the preparation of ECs [5,36,37]. Similar preferences for ceramic materials were observed by Wahab et al. [38] and Al Moaleem et al. [22], who noted the preference for ceramic materials among Jordanian and Saudi dentists, respectively. Zaki et al. [23] reported that 82.5% of participants believed ECs could be fabricated using pressable ceramic technology. However, our study found that 72.8% of participants preferred using CAD/CAM technology for EC fabrication. Regarding the fracture strength of EC restorations, Biacchi and Basting [39] demonstrated that ECs have significantly higher fracture strength compared to conventional crowns supported by glass fiber posts. In our study, 68.3% of participants agreed that ECs can serve as a substitute for post-and-core systems. Lin et al. [40] found that ECs transfer force to the pulp chamber’s wall and periodontal tissue rather than to the root canal wall and geometrically reduce the rotation center of the restoration compared to post-core and full crown systems. Despite this, 42% of participants in our survey noted that ECs might sometimes increase the incidence of tooth fractures. Ghoul et al. [6] found that 90% of EC failures were due to tooth fractures and restoration dislodgement occurring on the side opposite the stress.

Al Moaleem et al. [22] found that only 6% of participants had cemented more than 10 ECs, with the majority (42.02%) reporting they had never used ECs based on their training experience. This aligns with our findings, as most participants (64.5%) had never cemented ECs in their clinics. This is likely due to the prevalence of prefabricated post-core procedures, followed by ECs and cast post-core. During the cementation of ECs, a rubber dam was employed to ensure proper isolation. The tooth surface was etched with 37% phosphoric acid for 15 seconds, then rinsed and dried. A bonding agent was subsequently applied and polymerized with a curing light for 20 seconds [26]. In our study, 43.1% of participants used rubber dams during EC cementation. Postoperative radiographic evaluation is crucial for long-term success; in this study, about half of the participants (51.7%) performed post-cementation radiographs of ECs.

The difference in modulus of elasticity between dentine and ceramic can lead to debonding of the restoration and potential root fractures. Therefore, proper case selection is crucial for the long-term success of ECs [23]. Most dental professionals (80.0%) identified debonding as the primary failure mode for ECs [41]. This finding is consistent with our study, where 65.5% of participants also cited debonding as a major cause of EC failure. Based on the current study, dental practitioners generally demonstrate adequate theoretical knowledge and clinical performance regarding ECs. However, the varied responses suggest a need for a clear protocol outlining the indications, diagnosis, preparation, and cementation of ECs. This need is evident for both experienced and less experienced dentists, as regular application of these materials and procedures in practice is essential to align with the associated knowledge.

This study has several limitations, including a limited sample size from a single population and a short study duration.

## Conclusions

ECs can effectively replace conventional post-core-crown systems for restoring endodontically treated teeth, particularly when dealing with severely damaged dental tissues. Our study found that most participants demonstrated a good level of knowledge and practical experience with EC restorations. Therefore, incorporating ECs as a major topic in the postgraduate prosthodontics curriculum is recommended.

## Appendices

Appendix 1: Questionnaire

Section 1: Demographic data of the participants

Gender

Male

Female

Your level of education

Undergraduate

Intern

Graduate with less than five years of experience

Graduate with more than five years of experience

Graduation country

Libya

Arabic country

USA

European country

Other

Workplace

Government

University

Private

Unemployed

Section 2: Knowledge about endocrown

Q1: From where did you get the information about endocrown?

College

Workshop

Media

By email

Q2: Endocrown restorations are indicated in:

All teeth

Molar and premolar teeth

Molar teeth

I don't know

Q3: The goal of using endocrown is to achieve minimal invasive preparations and preserve the existing tooth structure.

True

False

I don't know

Q4: Which type of finish line is used during endocrown preparation?

Shoulder

Chamfer

Buttjoint

I don't know

Q5: The material of choice for endocrown is all ceramic.

Yes

No

I don’t know

Q6: Endocrowns are fabricated using:

CAD/CAM technique

Pressable technique

I don't know

Q7: The fracture resistance of endocrown is higher than that of conventional crowns.

True

False

I don’t know

Q8: The ideal extension of endocrown in the pulp chamber is:

2 mm

3 mm

4 mm

I don't know

Q9: If the floor of the pulp chamber is irregular, the failure of endocrown is increased.

True

False

I don’t know

Q10: Does endocrown increase the rate of tooth fracture?

Always

Never

Sometimes

I don't know

Q11: Can endocrown be a substitute for a conventional post, core, and crown?

Yes

No

I don't know

Section 3: Practice about endocrown

Q12: How many endocrowns did you cement in the clinic in previous years?

None

Less than 5

Between 6-10

More than 10

Q13: How often do you use a rubber dam during endocrown cementation?

Always

Sometimes

Never

Q14: How often do you use a radiograph to check the endocrown extension in the pulp chamber?

Always

Sometimes

Never

Q15: The most common "failure mode" of endocrown is debonding.

Yes

No

I don’t know

Q16: Tooth preparation steps to receive endocrown are more difficult than post, core and crown.

Yes

No

I don’t know

Q17: Tooth preparation and impression to receive endocrown are more time-consuming than post, core, and crown.

Yes

No

I don't know

Q18: The fracture resistance and bonding strength can be increased by the presence of an enamel margin.

Yes

No

I don't know
